# Impact of age on antigen-specific T lymphocyte response in patients treated with PSA-TRICOM

**DOI:** 10.1186/2051-1426-2-S3-P155

**Published:** 2014-11-06

**Authors:** Harpreet Singh, Ravi Madan, Jennifer Marte, Geraldine O'Sullivan-Coyne, Seth Steinberg, Christopher Heery, Jeffrey Schlom, James Gulley

**Affiliations:** 1 National Cancer Institute/ Laboratory of Tumor Immunology and Biology, Bethesda, MD, USA; 2 National Cancer Institute, Bethesda, MD, USA; 3 National Cancer Institute/ Genitourinary Malignancies Branch, Bethesda, MD, USA; 4 Natinal Cancer Institute/ Biostatistics and Data Management Section, Bethesda, MD, USA; 5 National Cancer Institute/ Center for Cancer Research, Bethesda, MD, USA

## Background

PSA-TRICOM is a vector-based therapeutic cancer vaccine designed to generate a targeted anti-tumor immune response against prostate-specific antigen (PSA)-expressing tumor cells. Early clinical trials have evaluated the impact of this vaccine on the immune system and demonstrated promising clinical activity. Although many patients with prostate cancer are advanced in age, little is known about how these shifts in immune phenotype affect responses to immunotherapies such as cancer vaccines.

## Methods

We conducted a broad overview of both published and new data to analyze the immune responses to PSA-TRICOM. Immune responses included ELISPOT for antigen-specific immune response and flow-cytometry analysis of peripheral immune cells.

## Results

104 patients with prostate cancer were tested for PSA-specific T lymphocyte responses to PSA-TRICOM. Age of patients ranged from 47-87 years, with a median of 66.3 years. Spearman correlation coefficient was used to interrogate an association between the age of patients and magnitude of antigen-specific T lymphocyte response. There was no association between age and immune response as measured by the ratio of maximum PSA spots/million to PSA spots/million prior to vaccine (r = 0.099; p = 0.32). Influenza responses were assessed by ELISPOT as standard controls, with no differences observed based on age. For most patients PSA-specific immune responses (likely memory cells) seen 28 days following the most recent vaccine are quantitatively similar to levels of circulating influenza-specific T cells in the same patients.

## Conclusion

There is minimal understanding about how responses to immune-based therapies vary with age. We found no evidence that the magnitude of immune responses generated by PSA-TRICOM is correlated with age of treated patients. This suggests that elderly patients may derive equal benefit from immune-based therapies as their younger counterparts. Further immune analysis continues in an ongoing Phase III of PSA-TRICOM in metastatic castration resistant prostate cancer (NCT01322490), currently accruing worldwide, and 2 trials combining PSA-TRICOM with Enzalutamide (biochemical recurrence/NCT01875250 and mCRPC/NCT01867333), which are currently accruing at the NCI.

